# Long-term effects of intravenous iloprost in patients with idiopathic pulmonary arterial hypertension deteriorating on non-parenteral therapy

**DOI:** 10.1186/1471-2466-11-56

**Published:** 2011-12-01

**Authors:** Lars Knudsen, Alexander Schurawlew, Nils Nickel, Henning Tiede, Hossein A Ghofrani, Heinrike Wilkens, Ralf Ewert, Michael Halank, Hans Klose, Carlos Bäzner, Jürgen Behr, Marius M Hoeper

**Affiliations:** 1 Department of Respiratory Medicine, Hannover Medical School, Hanover, Germany; 2 Medical Clinic II/V, University Hospital Giessen and Marburg, Justus-Liebig University, Giessen, Germany; 3 Department of Pneumology, University Hospital Homburg, Homburg/Saar, Germany; 4 Department of Cardiology and Respiratory Medicine, University Hospital Greifswald, Greifswald, Germany; 5 Department of Internal Medicine I, Carl-Gustav-Carus University, Dresden, Germany; 6 Department of Pneumology, University Hospital Hamburg-Eppendorf, Hamburg, Germany; 7 Division of Pulmonary Diseases, Department of Internal Medicine I, Ludwig Maximilans University, Klinikum Grosshadern, Munich, Germany; 8 Department of Internal Medicine III, Pneumology, University Hospital Bergmannsheil, Bochum, Germany

## Abstract

**Background:**

The majority of patients with idiopathic pulmonary arterial hypertension (IPAH) in functional classes II and III are currently being treated with non-parenteral therapies, including endothelin receptor antagonists (ERA), phosphodiesterase (PDE)-5 inhibitors, inhaled iloprost or combinations of these substances. If these treatments fail, current guidelines recommend the addition of parenteral prostanoid therapy. There is, however, limited evidence for the efficacy of parenteral prostanoids when added to combinations of non-parenteral therapies.

**Methods:**

In this retrospective, multicentre study we collected data from consecutive IPAH patients receiving intravenous iloprost in addition to optimized non-parenteral therapy between Jan 2002 and Dec 2009. Analyses included 6 min walk distance (6MWD), functional class, need for transplantation, and survival.

**Results:**

During the observation period, 50 patients were treated with intravenous iloprost in addition to non-parenteral therapy; 44% of the patients were on dual combination therapy and 52% on triple combination. Three months after initiation of iloprost, functional class had improved in 24% of the patients and the median 6MWD had increased from 289 m to 298 m (n.s.). During the observation period, 22 patients (44%) died and 14 (28%) underwent lung transplantation. The probabilities of LuTx-free survival at 1, 3 and 5 years following iloprost initiation were 38%, 17% and 17%, respectively. A 6MWD < 300 m and persistent functional class IV at 3 months after initiation of intravenous iloprost were predictors of an adverse outcome.

**Conclusion:**

In essence, late initiation of intravenous iloprost in IPAH patients who previously failed to respond to non-parenteral therapies appears to be of limited efficacy in the majority patients. Alternative therapeutic options are currently not available, underlying the need for the development of new drugs.

## Background

Less than 10 years ago, intravenous epoprostenol was the standard treatment for patients with idiopathic pulmonary arterial hypertension (IPAH). The first intravenous prostanoid studied in patients with IPAH, epoprostenol is still the only drug for which improved survival in treatment-naive patients with advanced IPAH has been demonstrated [1]. Newer prostanoids used for intravenous therapy are treprostinil and iloprost [2-5]. It is unclear whether the efficacy of both drugs is comparable to epoprostenol as this has not yet been formally evaluated in randomized placebo controlled or head-to-head comparison trials. The need for parenteral administration, however, is a major drawback for all of these drugs.

In recent years, non-parenteral therapies, especially endothelin receptor antagonists (ERAs), phosphodiesterase (PDE)-5 inhibitors and inhaled prostanoids have replaced intravenous prostanoids as preferred initial therapies for patients presenting in functional classes II or III [6-10]. Parenteral prostanoids are now mainly used in patients presenting with advanced disease or when the less invasive therapies have been exhausted, respectively.

If patients deteriorate while receiving optimized non-parenteral therapies it is advocated to add an intravenous prostanoid [11] although this approach has not undergone the scrutiny of carefully conducted clinical trials. In the present study we investigated the long-term effects of adding intravenous iloprost to oral therapies in patients with severe IPAH.

## Methods

The present study was performed at 7 German pulmonary hypertension referral centres. Data were collected retrospectively from consecutive adult patients with IPAH who had received intravenous iloprost in addition to optimized oral therapies (PDE-5 inhibitor and/or ERA) between Jan 1^st ^2002 and Dec 31^st ^2009. Oral therapy was a requirement for inclusion, whereas additional therapy with inhaled iloprost was facultative. Follow-up ended March 31^st ^2010. Patients with other forms of pulmonary hypertension were excluded. This analysis was approved by the institutional review boards of the participating centres and all patients gave written informed consent.

There was no overlap between the present patient population and the patients reported in an earlier paper on the effects of intravenous iloprost in patients failing inhaled iloprost therapy, as in the prior study, patients were not pre-treated with an ERA or PDE-5 inhibitor and initiation of intravenous iloprost was between 1997 and 2001 [3].

Data on hemodynamics, functional class and 6 min walking distance (6MWD) were collected from different time points: (i) the initial assessment at the time of diagnosis, (ii) the last assessment prior to initiation of intravenous iloprost therapy (baseline iloprost; BL-Ilo), (iii) 3 and (iv) 12 months thereafter. Hemodynamic data from right heart catheterization were obtained from all patients at the time of diagnosis, i.e. prior to initiation of PAH-targeted therapy. For the assessment of hemodynamics prior to BL-Ilo, catheter data were only considered when they were not older than 3 months.

The patients enrolled in this analysis were treated according to local standards with respect to oxygen-supplementation, diuretics, anticoagulation and non-parenteral therapies with ERAs, PDE-5 inhibitors and inhaled iloprost.

There were no predefined criteria for the initiation for intravenous iloprost treatment. Decisions on start, dosage and up-titrations as well as follow-up visits were made by the responsible physicians at the participating centres. All patients were hospitalized for initiation of therapy. Iloprost was diluted in saline to a volume of 100 ml and administered via a port- or Hickman-catheter using a portable pump (CADD-1; Deltec, St. Paul, MN, USA). The medication was prepared under sterile conditions by specialized pharmacists and delivered to the patients' homes. The cassettes containing the medication were replaced every 48 hrs. The iloprost dose was titrated according to individual tolerability and clinical effects. The general aim was to reach the highest tolerated dose of intravenous iloprost.

### Statistical evaluation

Data were collected on Microsoft office Excel 2007 spreadsheets. Survival analyses were carried out using GraphPad PRISM 5.0 for Windows (GraphPad Software Inc., Software MacKiev).

Pairwise statistical comparisons between baseline parameters at diagnosis (Dx) and BL-Ilo were performed using a t-test if the data were normally distributed according to the Kolmogorov-Smirnov-test. Otherwise a nonparametric U-test was carried out. To compare the data obtained at month 3 and 12 of intravenous iloprost therapy with those obtained at BL-Ilo, multiple comparisons were performed by repeated one-way ANOVA followed by Tukey's correction for multiple comparisons.

Kaplan-Meier curves were plotted for overall survival with censoring of patients who had undergone lung transplantation (LuTx) and for LuTx-free survival (i.e., combined endpoint, death or transplantation; Tx-free survival). Survival analyses were performed from the time of diagnosis and from the time when intravenous iloprost therapy was started.

The probability of survival for each patient at the time t after diagnosis was calculated applying the National Institute of Health (NIH) equation [12,13].

$$\text{p}\left(\text{t}\right)\;=\text{ H}{\left(\text{t}\right)}^{\text{A}\left(\text{x},\text{y},\text{z}\right)}.$$

H(t) was defined by the time t via H(t) = 0.88-0.14t+0.01t^2 ^and A(x, y, z) could be calculated from the results of the RHC: A(x, y, z) = e^0.007325x+0.0526y-0.3235z^, where x was the mean pulmonary arterial pressure (P_PA_), y was the mean right atrial pressure (P_RA_) and z was the cardiac index (CI). The expected value for each patient to be alive at different time points was determined and the expected Kaplan-Meier curve was plotted and compared to the observed Kaplan-Meier survival curves for overall survival and LuTx-free survival. A log-rank test (= Mantel-Cox test) was performed to compare the observed and expected survival curves for significant differences.

In order to identify risk factors for an adverse outcome (death or LuTx), an univariate Cox-regression analysis was performed using the time interval between diagnosis and initiation of i.v. iloprost as a dichotomized variable at the median, hemodynamic parameters, functional class and 6 min walking distance at BL-Ilo and 3 months later (SPSS version 18.0, Inc., Chicago, Illinois, USA).

Depending on functional class (III or better vs. IV) and the 6MWD (cut-off 300 m) at 3 months, Kaplan-Meier survival curves of subgroups were plotted and compared, using a log-rank test (Mantel-Cox test). The cut-off of 300 m was chosen as this value was close to the median of 298 m 3 month after BL-ilo.

A correlation analysis comparing dosages of Iloprost at 3 months and changes in 6MWD was performed using the Spearman test.

Results are shown as median and interquartile range (IQR), unless stated otherwise. A p-value < 0.05 was considered statistical significant for all analyses.

## Results

Between January 1^st ^2002 and December 31^st ^2009, fifty patients with IPAH were treated with intravenous iloprost in addition to optimized non-parenteral therapy (see Table 1 for details). The median interval between diagnosis and BL-Ilo was 34 months, ranging from 1 to 104 months. Compared to the time of diagnosis, at BL-Ilo there was a deterioration in functional class and a significant decrease in the 6MWD (Table 1). Hemodynamic assessment demonstrated a significant increase in P_RA _while the remaining hemodynamic parameters were virtually unchanged (Table 1).

**Table 1:** Patient characteristics

Time point	Dx	Last measurement prior to start of IV iloprost
**Subjects (n)**	50	50

**Age (years)**	42 (29-58)	44 (31-63)

**Interval diagnosis and initiation of iloprost (month)**		34 (20-47)

**Female/male**	34/16	34/16

**NYHA class II/III/IV**	7/30/13	1/24/24

**6MWD (m)**	332 (229-434)	289 (132-370) ^#^

**PAH-specific therapy**	Initial therapy	
Sildenafil alone	11 (22%)	0 (0%)
ERA alone	22 (44%)	2 (4%)
Inhaled Iloprost alone	8 (16%)	0 (0%)
Sildenafil + ERA	6 (12%)	14 (28%)
Sildenafil + inhaled Iloprost	3 (6%)	3 (6%)
ERA + inhaled Iloprost	0 (0%)	5 (10%)
Triple therapy	0 (0%)	26 (52%)

**Hemodynamics**	n = 50	n = 26
P_RA _(mmHg)	7 (3-13)	12 (7-16) ^#^
P_pa _(mmHg)	59 (48-68)	56 (49-65)
P_pcw _(mmHg)	7 (5-9)	9 (7-11)
CI (L/min/m^2^)	2.0 (1.5-2.3)	1.7 (1.4-1.9)
PVR (dyn*s*cm^-5^)	1, 251 (852-1, 705)	1, 205 (954-1, 499)
S_V_O_2 _(%)	58 (54-66)	58 (54-69)

Characteristics at diagnosis (Dx) and start of intravenous iloprost. Absolute values are given regarding PAH-specific therapy, functional class and gender. Age, interval from Dx to BL-ilo, 6MWT and hemodynamic parameters are given as median (interquartile range; IQR). #: p < 0.05 vs Dx.

The majority of patients (78%) received oral medications (sildenafil, an ERA or a combination of sildenafil and ERA) as first-line therapies; 16% started monotherapy on inhaled iloprost and 6% a combination of sildenafil and inhaled iloprost. At BL-Ilo, all patients received at least one oral agent, i.e. an ERA or sildenafil, and the majority of patients (80%) were receiving a combination of both substances. Forty-three patients (86%) received sildenafil at a median dose of 150 mg/d (IQR, 100-180 mg/d). Forty-seven patients (94%) received an ERA, 36 bosentan, 9 sitaxentan and 2 ambrisentan at median daily dosages of 250 mg, 100 mg and 10 mg, respectively. In addition, 68% of the patients were treated with inhaled iloprost (30 μg per day). Hence, at BL-Ilo, 52% of the patients were receiving triple combination therapy and another 44% double combination therapy (Table 1).

Oral therapies were continued in all cases when intravenous iloprost was started. At that point in time inhaled iloprost was withdrawn in 14 patients and continued in the remaining 20 patients.

The median dose of intravenous iloprost was 1.73 ng/min/kg (IQR, 1.14-2.18 ng/min/kg) after 3 months and 1.80 ng/min/kg (IQR, 1.20-2.40 ng/min/kg) after 12 months, respectively.

### Effect of intravenous iloprost therapy on functional class

At the time of diagnosis, 14% of the patients were in functional class II, 60% in functional class III and 26% in functional class IV. At BL-Ilo, one patient (2%) was in functional class II, while 49% each were in functional class III and IV, respectively.

Three months after iloprost was started, 7 patients had died and 3 had undergone LuTx. Functional class data were available from 38 out of the remaining 40 patients. Nine of these patients (24%) showed an improvement in functional class. Two patients (5%) deteriorated from functional class III to functional class IV and 27 patients (71%) remained in the pre-treatment functional class.

At 12 months after BL-Ilo, 18 patients (36%) had died and 10 (20%) had undergone LuTx. Sixteen patients were within the observational period. Compared to the assessment done at BL-Ilo, none of the remaining 16 patients showed further improvement of the functional class; two patients had deteriorated from class III to IV and 14 remained in the same class as before.

### Effect of intravenous iloprost therapy on 6 min walking distance

**Figure 1:**
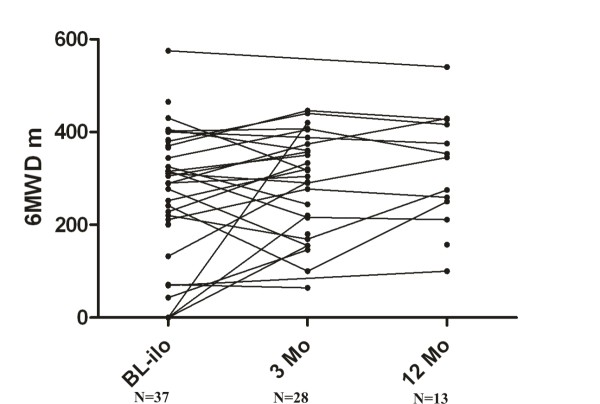
**Available data of 6MWD for each patient at different time points e.g. start of iloprost (BL-ilo), 3 months (3 Mo) and 12 months (12 Mo) after iloprost initiation**. The median of the 6MWD increased from BL-ilo to 3 Mo and 12 Mo (289 m vs. 298 m vs. 345 m). However, the between group differences were not statistical significant (one-way ANOVA: p = 0.09)

Individual data on 6MWD are shown in Figure 1. Those data were available for 37 patients at BL-Ilo. After 3 and 12 months, 6MWD data were available from 28 and 13 patients, respectively. Compared to BL-Ilo, the median 6MWD increased slightly after 3 months of therapy from 289 m (IQR, 132-370 m) to 298 m (IQR, 189-171 m). For those patients for which 6MWD were available after 12 months, there was a further increase to 345 m (IQR, 231-422 m); however, none of these changes were statistically significant. No significant correlation was found between the dosage of intravenous iloprost and the change in 6MWD at 3 months compared to BL-Ilo (r = 0.33, p = 0.08; data not shown).

### Survival and lung transplantation

During the observation period, 22 patients (44%) died and 14 (28%) underwent lung transplantation. Fourteen patients (28%) survived until the end of the study. The median LuTx-free survival was 45 months (IQR, 30-92 months) from the time of diagnosis and 9 months (IQR, 4-25 months) from BL-Ilo. The median overall survival (patients undergoing LuTx censored at the time of transplantation) was 87 months (IQR, 39-109 months) after diagnosis and 15 months (IQR, 7-76 months) after BL-Ilo.

### Comparison of expected and observed survival after diagnosis

**Figure 2:**
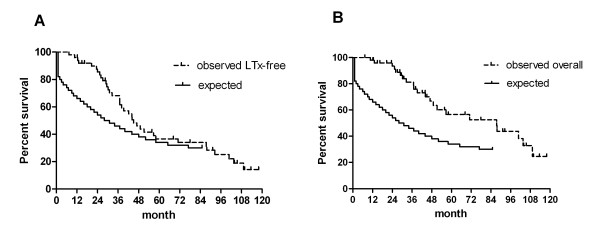
**Kaplan-Meier curves showing the expected survival in comparison to the observed LuTx-free (A) and observed overall (LuTx censored) (B) survival from the time of diagnosis**. Patients at risk were n = 50, n = 47, n = 41, n = 32, n = 21, n = 16 and n = 14 at diagnosis, 12, 24, 36, 48, 60 and 72 months after diagnosis (observed survival). Level of significance: LuTx-free survival vs. expected survival: p = 0.15. Overall survival vs. expected survival: p < 0.01. |: censored patients.

Figure 2 shows the observed LuTx-free (Figure 2a) and the observed overall survival (Figure 2b) after diagnosis as well as the expected survival as derived from the NIH registry equation [12]. The observed median LuTx-free survival and the overall survival after diagnosis were 45 and 87 months, respectively. The expected median survival after diagnosis was 30 months which was significantly lower than the observed overall survival (p < 0.01). The observed LuTx-free survival rates 1, 2, 3, 4, 5 and 6 years after diagnosis were 96%, 86%, 68%, 46%, 37% and 34% respectively. The corresponding overall survival rates after diagnosis were 98%, 93%, 81%, 67%, 57% and 53%, respectively. In comparison, the expected survival rates were 66%, 54%, 46%, 38%, 34% and 32%, which was significantly lower than the observed overall survival rates (p < 0.01).

### Comparison of expected and observed survival after initiation of intravenous iloprost

**Figure 3:**
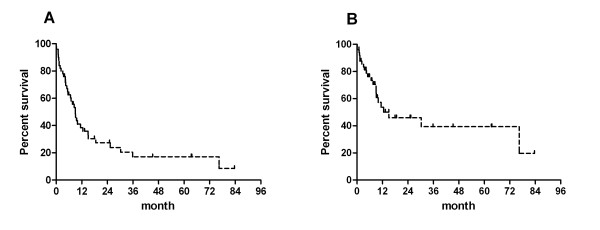
**Kaplan-Meier curves showing the observed LuTx-free (A) and observed overall (B) survival from initiation of continuous intravenous iloprost**. Patients at risk n = 50, n = 16, n = 10, n = 6, n = 5, n = 5, n = 3 at BL-ilo, 12, 24, 36, 48, 60 and 72 months after initiation of iloprost therapy (observed survival). |: censored patients.

Figure 3 shows the Kaplan-Meier curves of the LuTx-free survival (Figure 3a) and the overall survival (Figure 3b) following the initiation of continuous intravenous iloprost therapy.

The probabilities for LuTx-free survival at 1, 2, 3, 4, 5 and 6 years were 38%, 27%, 17%, 17%, 17% and 17%, respectively. In other words, 62% of these patients either died or underwent LuTx within one year after initiation of intravenous iloprost therapy. The observed overall survival rates at these time points were 54%, 46%, 39%, 39%, 39% and 39%. The median LuTx-free survival was 9 months whereas the median overall survival was 15 months.

### Predictors of survival

None of the parameters assessed at BL-Ilo was significantly associated with survival by means of univariate Cox regression analysis (data not shown). Patients in functional class IV at BL-Ilo had a median LuTx-free survival of 6 months which did not differ significantly from the 11 months seen in patients in functional class III or better (p = 0.23, hazard ratio 1.5, 95% CI 0.77-3.08).

Comparing survival curves of patients according to the 6MWD being more or less than 300 m did also show no significant differences (data not shown). The median LuTx-free survival was 11 months in patient walking > 300 m at BL-Ilo compared to 10 months in patients walking < 300 m (p = 0.78, hazard ratio 1.13, 95% CI 0.49-2.56).

**Figure 4:**
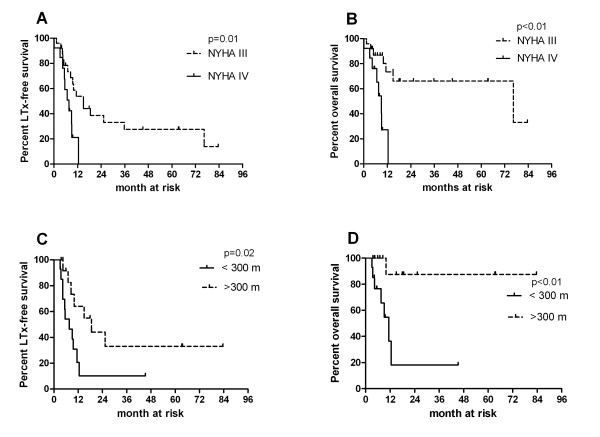
**Kaplan-Meier curves demonstrating the impact of functional class (A and B) and 6MWD (C and D) 3 months after introduction of intravenous iloprost on the LuTx-free and overall survival from the time point iloprost-start**. LuTx-free survival: Hazard ratio NYHA IV vs. III 3.87; 95% CI 1.38-10.84; Hazard ratio 6MWD < 300 m vs. > 300 m 3.14, 95% CI 1.17-8.45. Overall survival: Hazard ratio of NYHA IV vs. NYHA III: 8.39, 95% CI 2.28-30.83; Hazard ratio of 6MWT < 300 m vs. > 300 m: 8.76, 95% CI 2.05-37.5. |: censored patients.

In contrast, the parameters obtained at 3 months after initiation of intravenous iloprost treatment were more useful in predicting outcomes (Figure 4a-d). All patients who were in functional class IV after 3 months of therapy died within one year. In contrast, patients in functional class III after 3 months of therapy had a median LuTx-free survival of 15 months (p = 0.01, hazard ratio 3.87, 95% CI 1.38-10.84).

Patients with a 6MWD < 300 m at month 3 had a median LuTx-free survival of 8 months, whereas those walking > 300 m had a significantly better prognosis with a LuTx-free median survival of 19 months (p = 0.02, hazard ratio 3.14; 95% CI 1.17-8.45).

### Line infections

In this cohort, 9 line infections were observed in 7 patients accounting for 0.41 cases of line infections per 1, 000 days of treatment.

## Discussion

The present study casts doubts about the efficacy of intravenous iloprost at least in the majority of cases when added to IPAH patients who failed to respond adequately to non-parenteral therapies. Only a small subgroup of patients seemed to benefit from the intravenous iloprost therapy. The overall survival rates 1, 3 and 5 years after diagnosis were 98%, 81%, and 57% which is in accordance with outcome data reported from other series [14-16]. However, once these patients failed non-parenteral therapy, adding intravenous iloprost had apparently little effects on 6MWD, functional class, and survival. It is not possible to fully appraise the effects of intravenous iloprost as there was no control group but the fact that 62% of the patient died or required LuTx within one year after this treatment was initiated is troublesome. The worst outcome was seen in patients remaining in functional class IV and/or having a 6MWD < 300 m after intravenous iloprost therapy had been initiated.

The present data are in agreement with an earlier report on the effects of intravenous iloprost as rescue therapy in patients failing inhaled iloprost treatment [3]. In fact, the observed functional improvements as well as the survival rates after initiation of intravenous iloprost were even worse in the present series (e.g., LuTx-free survival after 12 months 38% vs. 57% in the previous series). The apparent poorer response of clinical and functional parameters in the present study is most likely a consequence of the pre-existing exhausted non-parenteral therapy.

Though parenteral prostanoids are used as salvage treatment for patients failing other forms of therapy, there is limited data to support this recommendation. None of the available parenteral prostanoids has been systematically studied as add-on therapy to non-parenteral combination therapy. The available data is currently limited to non-controlled data from single centre studies and most of these studies have used intravenous epoprostenol [17-19].

In a retrospective analysis of 36 patients receiving the endothelin receptor antagonist bosentan, add-on administration of intravenous epoprostenol (n = 30) or inhaled iloprost (n = 6) led to a significant increase in 6MWD (347 m vs 310 m, p = 0.031) associated with an improvement in functional class in 39% of cases [17]. The mean exposure to bosentan was 12 ± 10 months prior to prostanoid therapy. The 1 and 2 year-survival rates after initiation of prostanoid therapy were 88% and 71%, respectively [17].

Another series reported on 16 patients with IPAH who received either intravenous epoprostenol (n = 6) or subcutaneous (s.c.) treprostinil (n = 10) in addition to bosentan monotherapy (n = 6) or bosentan/sildenafil combination therapy (n = 10). The 6MWD at start of prostanoid therapy was 363 ± 27 m and the mean interval between start of oral therapy and start of prostanoids was 21 ± 5 months. Improvements in 6MWD after 4 months were 86 m in the bosentan group versus 41 m in the bosentan/sildenafil group. The difference between both groups was not statistically significant. During a median observation period of 18 months, there was only one death and autopsy revealed that this patient suffered from pulmonary veno-occlusive disease [18].

Finally, a series of 23 patients from Australia reported on the use of epoprostenol as salvage therapy in patients failing oral therapies (mostly monotherapy with either bosentan or sildenafil; details and treatment durations were not stated). In contrast to the previously discussed series, epoprostenol was not added to the oral therapies but replaced them. The reported improvement in 6MWD was 117 m (70-264 m; p = 0.002); three patients died and 9 required transplantation [19].

It is difficult to compare these series with our data as patient populations, selected therapies and treatment durations varied substantially. The majority of patients in the present study were on double or triple combination therapy and the median duration of non-parenteral therapy was 34 months, i.e. longer than in the other series. However, all other series reported on more substantial functional improvements and apparently better survival rates associated with parenteral prostanoid therapy. It is possible that these differences resulted from earlier use of parenteral therapy and/or from the fact that non-parenteral therapy had not been fully exhausted. However, another possible explanation for the observed differences may be differences in efficacy between iloprost and epoprostenol [20].

Although previous studies suggest similar effectiveness of iloprost and epoprostenol [21,22], these two drugs have never been formally compared in randomized clinical trials. Intravenous iloprost, in fact, has never been thoroughly studied in patients with IPAH and the fact that it is widely used in Germany and some other European countries is related to the lack of approval of epoprostenol and some other historical reasons. It cannot be ruled out that differences regarding effectiveness in IPAH exist. Disturbing findings of the present study are not only the low survival rates but also the apparent lack of lasting clinical improvements in the vast majority of our patients. It will be difficult, if not impossible, to perform prospective large-scale trials comparing intravenous epoprostenol and intravenous iloprost. A possible way to derive some data addressing the question whether epoprostenol is or is not more efficacious than iloprost might be to switch patients not responding sufficiently from intravenous iloprost to epoprostenol.

We cannot rule out insufficient dosing of iloprost as a contributing factor in the present study. The mean iloprost dose at 12 months (1.9 ng/min/kg) was lower than in our previous study (2.6 ng/min/kg after 6 months) [3]. However, all centres attempted to up titrate iloprost to the highest tolerated dose and there was no association between iloprost dose and changes in 6MWD.

One may also argue that intravenous iloprost therapy was initiated too late in the present population. Earlier initiation of therapy is likely to be associated with better survival rates but it is unclear whether this is caused by length bias or by a true survival effect. So far, no study has shown that earlier use of parenteral prostanoid therapy in patients with IPAH results in better long-term outcome.

Complications related to the delivery system appeared to be similar to other series and are therefore unlikely to be responsible for the present findings. In our cohort we observed 0.41 cases of line infections per 1, 000 days of treatment which is in line with previous studies reporting a rate of catheter-infections per 1, 000 treatment-days between 0.26 [23] and 0.42 [24], respectively, for epoprostenol, and 1.13 for treprostinil [24].

The present study has several important limitations including the relatively small number of patients, the lack of a control group, the retrospective design and the abundance of missing data. In addition, there was no formal study protocol and no pre-established criteria for starting and dosing intravenous therapy. All these limitations reflect the fact that our data was not derived from a prospective clinical trial but from clinical experience. Therefore, despite lacking robustness, our data represent real-life scenarios.

## Conclusion

The clinical effect of adding intravenous iloprost to IPAH patients failing on extensive non-parenteral therapy appears to be modest, and failed to prevent death or transplantation in the majority of patients. Despite all limitations, our data suggest that the role of i.v. prostanoid therapy in the era of oral and inhalative treatments for IPAH needs to be redefined. In pre-treated patients timing and choice of i.v. prostanoid therapy needs to be reconsidered and should be further addressed in dedicated clinical trials. Finally, these results underline the need for the development of new agents out of new classes of drugs in this therapeutic indication.

## Competing interests

LK has no conflict of interest to disclose

AS has no conflict of interest to disclose

NN has no conflict of interest to disclose

HT has received fee for speaking by BayerSchering

HAG has received fees for speaking at conferences and/or consultations from Actelion, Bayer, Gilead, GSK, Lilly, LungRx, Novartis and Pfizer

HW received honoraria for speaking at conferences and/or consultations from Bayer-Schering, Pfizer, Actelion, GlaxoSmithkline and United Therapeutics

RW has no conflict of interest to disclose

MH has received honoraria from Actelion, Bayer Schering Pharma AG, GlaxoSmithKline, Lilly, Novartis, Pfizer and United Therapeutics; has received travel grants from Actelion, Bayer Schering Pharma AG, Lilly, Novartis and Pfizer; and has participated in advisory board activities for GSK, Lilly and United Therapeutics.

HK has received fees for educational lectures, travel grands to ATS 2008 and a study nurse was supported be Bayer Schering. HK got financial grants from: Actelion pharmaceuticals, Bayer Schering, Glaxo Smith Kline, Pfizer, United Therapeutics, Novatis Pharma.

CB has no conflict of interest to disclose

JB has received fees for speaking from Actelion, Altana, Bayer-Schering, Boehringer-Ingelheim, Encysive, GSK, Pfizer, Lilly, Nycomed. JB has served as consultant/advisor for Actelion, Bayer-Schering-Pharma, Lilly, Pari-Pharma, GSK, Pfizer. JB has received research grants from Actelion, Bayer-Schering, Pari-Pharma.

MMH has received fees for speaking at conferences and/or consultations from Actelion, Bayer, Gilead, GSK, Lilly, LungRx, Novartis and Pfizer

## Authors' contributions

All authors read and approved the final version of this manuscript. All authors were involved in interpretation and discussion of the results. LK wrote parts of the manuscript including "Methods" and "Results" and is responsible for the statistical analysis and the figures. AS collected data from patients' files at Hannover Medical School. NN carried out the univariate Cox regression analysis and was also involved the statistical analysis. HT and HAG were responsible for data collection from patients' files at Giessen University Hospital. HW was responsible for data collection from patients' files at the University Hospital Homburg, Saarland. RE was responsible for data collection from patients' files at the University Hospital Greifswald. MH was responsible for data collection from patients' files at the University Hospital Dresden. HK was responsible for data collection from patients' files at the University Hospital Hamburg. CB and JB were responsible for data collection from patients' files at the University Hospital Munich. MH wrote parts of the manuscript including "Introduction" and "Discussion" and was involved in designing the study.

## Pre-publication history

The pre-publication history for this paper can be accessed here:

http://www.biomedcentral.com/1471-2466/11/56/prepub
